# Implicating Causal Brain Magnetic Resonance Imaging in Glaucoma Using Mendelian Randomization

**DOI:** 10.3389/fmed.2022.956339

**Published:** 2022-07-01

**Authors:** Kangcheng Liu, Pengfei Wu, Bolin Chen, Yingjun Cai, Ruolan Yuan, Jing Zou

**Affiliations:** ^1^Eye Center of Xiangya Hospital, Central South University, Changsha, China; ^2^Hunan Key Laboratory of Ophthalmology, Changsha, China; ^3^National Clinical Research Center for Geriatric Disorders, Xiangya Hospital, Central South University, Changsha, China; ^4^Jiangxi Clinical Research Center for Ophthalmic Disease, Jiangxi Research Institute of Ophthalmology and Visual Science, Affiliated Eye Hospital of Nanchang University, Nanchang, China; ^5^Hunan Key Laboratory of Medical Genetics, School of Life Sciences, Central South University, Changsha, China

**Keywords:** diffusion tensor imaging, white matter, region-of-interest, glaucoma, Mendelian randomization

## Abstract

**Background:**

Glaucoma is hypothesized to originate in the brain but manifests as an eye disease as it possesses the common features of neurodegeneration diseases. But there is no evidence to demonstrate the primary brain changes in glaucoma patients. In the present study, we have used Mendelian randomization (MR) to understand the causal effect of brain alterations on glaucoma.

**Methods:**

Our MR study was carried out using summary statistics from genome-wide associations for 110 diffusion tensor imaging (DTI) measurements of white matter (WM) tracts (17,706 individuals), 101 brain region-of-interest (ROI) volumes (19,629 individuals), and glaucoma (8,591 cases, 210,201 control subjects). The causal relationship was evaluated by multiplicative random effects inverse variance weighted (IVW) method and verified by two other MR methods, including MR Egger, weighted median, and extensive sensitivity analyses.

**Results:**

Genetic liability to fornix fractional anisotropy (FX.FA) (OR = 0.71, 95%CI = 0.56–0.88, *P* = 2.44 × 10^–3^), and uncinate fasciculus UNC.FA (OR = 0.65, 95%CI = 0.48–0.88, *P* = 5.57 × 10^–3^) was associated with a low risk of glaucoma. Besides, the right ventral diencephalon (OR = 1.72, 95%CI = 1.17–2.52, *P* = 5.64 × 10^–3^) and brain stem (OR = 1.35, 95%CI = 1.08–1.69, *P* = 8.94 × 10^–3^) were associated with the increased risk of glaucoma. No heterogeneity and pleiotropy were detected.

**Conclusion:**

Our study suggests that the fornix and uncinate fasciculus degenerations and injures of the right ventral diencephalon and brain stem potentially increase the occurrence of glaucoma and reveal the existence of the brain-eye axis.

## Introduction

Glaucoma is one of the common blinding disorders characterized by progressive damage to the retinal ganglion cells (RGCs) along with visual field loss (1). Many elements are considered major risk factors, including intraocular pressure (IOP), genes, family factors, vascular factors, and high myopia (2, 3). However, elevated IOP is the only modifiable risk factor; thus, IOP reduction is considered a helpful treatment until now (4, 5). However, whereas many normal IOP individuals suffer from the disorder, IOP reduction could not completely stem the glaucoma progression (6). This suggests that it is necessary to find other effective ways to alleviate glaucomatous neurodegeneration.

The eye is the extension of the brain and has many embryological, functional, and developmental similarities with the central nervous system (7). As the hallmark of glaucoma, the primary cells to die in glaucoma are RGCs, which are typical neurons (8). Although the cell bodies of RGCs lie in the retina, most portions of axons lie outside the ocular area, which forms the optic nerve, optic chiasm, and optic tract (8). Besides, many observational studies have shown that glaucoma patients had cognition and memory decline (9, 10). In addition, motor coordinating impairment and psychological disorders such as depression and anxiety were prevalent among the glaucoma patients (11, 12). Based on these theories, various scholars hypothesize that brain tissues are associated with glaucoma pathogenesis (13, 14).

Magnetic resonance imaging (MRI) studies are an important tool as a non-invasive method to evaluate the structure, function, and neurochemistry of the brain (15). Some researchers investigated the structural and functional brain changes in patients with glaucoma using MRI. They reported white matter structural abnormalities in different parts of the visual pathway and altered brain connectivity beyond the visual system (16, 17). These observations led to the question of whether brain changes precede or follow the RGCs degeneration. Although an anterograde tans-synaptic degeneration might be responsible for some visual pathway damage, the brain structures outside the visual pathway generally reflect a primary neuropathological process. Besides, due to the high cost and limited accessibility to MRI facilities, it is hard to complete randomized, controlled trials to explore the primary brain changes in glaucoma patients.

Mendelian randomization (MR) uses a genetic variation to evaluate the inter-causality of disease risk factors, which is not affected by most acquired confounding factors (such as environment) (18). Thus far, MR has been used to derive the causal relationship between kidney damage and brain cortical structure, brain structures, and neuropsychiatric disorders, including Alzheimer’s disease (AD) (19, 20). Recently, a GWAS study reported an association of single nucleotide polymorphisms (SNPs) of brain structural measurements with glaucoma (21–24). These studies bought insight into finding the causal relationship between glaucoma and brain structures. Thus, we aimed to identify the causal relationship between white matter (WM) structures [diffusion tensor imaging (DTI), brain subregion volumes region of interest (ROI)], and glaucoma by using the two-sample MR analysis. Our results indicate the potential risks of glaucoma in the brain and provide new insight into the possible existence of the brain-eye axis.

## Materials and Methods

### Assessment of Assumptions

**FIGURE 1:**
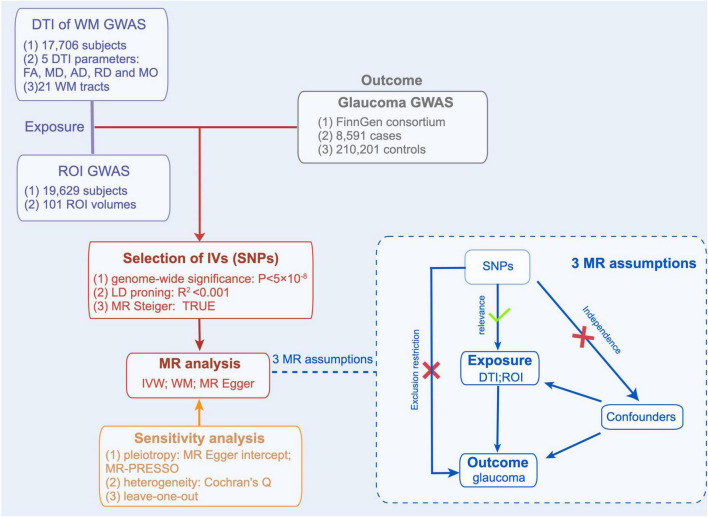
Mendelian Randomization Analysis Overview. Overview of Mendelian randomization analyses and major assumptions. DTI, indicates diffusion tensor imaging; WM, white matter; ROI, region of interest; FA, fractional anisotropy; MD, mean diffusivity; AD, axial diffusivity; RD, radial diffusivity; MO, mode of anisotropy.

The valid genetic instrumental variables (IVs) in the MR study must fulfill the 3 assumptions (Figure 1): (1) The IVs must be associated with DTI parameters of WM tracts and ROI volumes. (2) The IVs must not be associated with factors that confound the relationship between DTI parameters of WM tracts and glaucoma, ROI volumes, and glaucoma. (3) The IVs are only associated with glaucoma through DTI parameters of WM tracts and ROI volumes.

### The Data Source for Diffusion Tensor Imaging Measurements

The genetic variants of DTI parameters of WM traces were taken from the genome-wide association studies (GWAS) of Zhao et al. (21). This GWAS study consisted of a total of 110 DTI parameters from 17,706 subjects of European ancestry, including fractional anisotropy (FA), mean diffusivity (MD), axial diffusivity (AD), radial diffusivity (RD), mode of anisotropy (MO), and the average values of 21 WM tracts. The 21 WM tracts were labeled through the Enhancing NeuroImaging Genetics through Meta-Analysis (ENIGMA) DTI pipeline (22, 23), and the full names of the tracts are shown in Supplementary Table 1.

### The Data Source for the Region-of-Interest Volumes

The genetic variants of the ROI volumes came from the genome-wide association studies (GWAS) study of Zhao et al. (24). This GWAS study analyzed MRI data from 19,629 European participants through consistent procedures *via* normalization tools (ANTs)^1^ and used Mindboggle-101 atlas for labeling.

### Selection of Genetic Instruments

The single nucleotide polymorphisms (SNPs) related to DTI and ROI were screened to obtain the required IVs. First, the SNPs associated with DTI or ROI exhibited genome-wide significance (*P* < 5 × 10^–8^). Also, SNPs with linkage disequilibrium (LD) were removed (*R*^2^ < 0.001) in the LD data from the 1,000 Genomes Project (25). In addition, we removed the SNPs with the presence of palindromes. Finally, to clarify the direction of causality, all SNPs were examined using MR Steiger (26), and the SNPs that could lead to causality were removed.

### The Data Source for Glaucoma

To avoid the overlapping between DTI and ROI-related and glaucoma-related data sources as much as possible, the association of glaucoma relative SNPs was obtained from the FinnGen consortium (Release 5, May 11, 2021, comprising 218,792 individuals). This study defines glaucoma by H40-H42 of the International Classification of Disease-10 (ICD-10). Genotype data from the FinnGen research project included 8,591 glaucoma patients of Finnish origin and 210,201 control subjects.

### Sensitivity Analysis

To test the robustness of the MR estimates, we applied sensitivity analysis. First, MR Egger’s intercept values were used to assess the multiplicity of SNPs. The closer the intercept to 0, the lower the multiplicity was considered. Meanwhile, MR-Pleiotropy RESidual Sum and Outlier (PRESSO) (27) was used to evaluate further the SNPs pleiotropy that has the potential causality, and the SNPs with abnormalities were removed. Meanwhile, Cochran’s Q test was used for the assessment of heterogeneity. Finally, to ensure the robustness of the results, the analysis was carried out using the leave-one-out test.

### Statistical Method

“Two Sample MR” packages were used to estimate the MR results using the R software (version 1.4.1717). The Wald ratio was used to assess the causal effect of individual SNPs. Provided that sensitivity analysis was passed, inverse variance weighting (IVW) was used as the main method to assess MR effects. The weighted median (28) and MR-Egger regression (27) were used as additional methods to determine the MR results validity further. *P* < 0.05 was considered to have potential causality.

## Results

### Instrumental Variables Selection

A total of 394 SNPs were selected to predict 81 DTI parameters of WM traces genetically, and 389 SNPs were used to predict 61 ROI volumes (Supplementary Tables 2, 3). An overview of the flow charts is shown in Figure 1.

### Causal Associations Between Diffusion Tensor Imaging and Glaucoma

**FIGURE 2:**
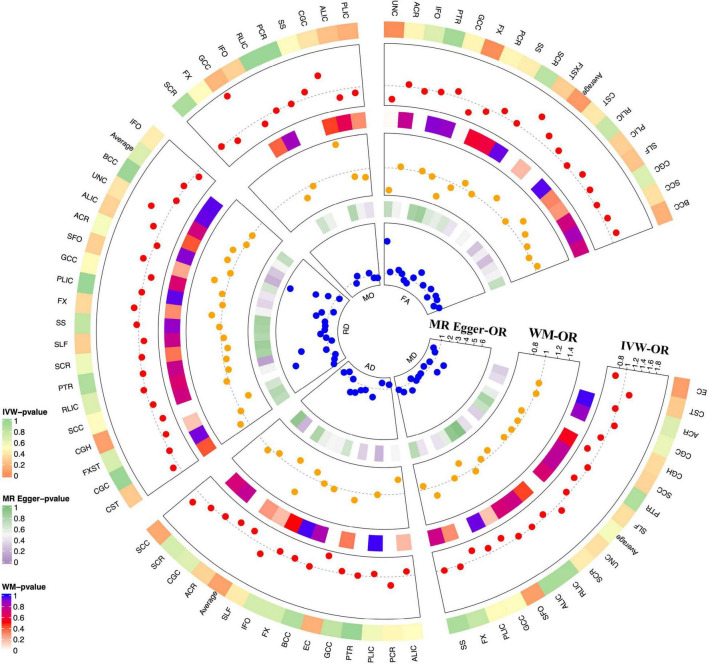
MR results for DTI-Glaucoma associations. Each radius represents OR of one DTI measurement (red dot: MR-IVW; yellow dot: orange dot; blue dot: MR-Egger). From outer to inner layer: *p*-value for MR-IVW; OR for MR-IVW; *p*-value for MR-WM; OR for MR-WM; *p*-value for MR Egger; OR for MR-Egger. The dotted gray line indicated OR = 1.

We evaluated the DTI-Glaucoma association by Mendelian randomization analysis, and the specific statistical results are shown in Figure 2 and Supplementary Table 4. There were two FA parameters of the white matter that reached statistical significance: fornix fractional anisotropy (FX.FA) and uncinate fasciculus fractional anisotropy (UNC.FA) (Table 1). Then, IVW was used to confirm that FX.FA (OR = 0.71, 95%CI = 0.56–0.88, *P* = 2.44 × 10^–3^), UNC.FA (OR = 0.65, 95%CI = 0.48–0.88, *P* = 5.57 × 10^–3^) was associated with a low risk of glaucoma. Additionally, we further validated the results of IVW by weighted median (FX.FA: *P* = 1.03 × 10^–2^; UNC.FA: *P* = 2.42 × 10^–2^) and MR-egger (FX.FA: *P* = 0.614, UNC.FA: *P* = 0.735) (Table 1).

**TABLE 1:** MR results between WM and glaucoma.

WM	Method	SNP	OR	95%CI	*P*-value
FX.FA	IVW	7	0.71	0.56–0.88	2.44 × 10^–3^
FX.FA	WM	7	0.69	0.52–0.92	1.03 × 10^–2^
FX.FA	MR egger	7	0.64	0.13–3.23	0.614
UNC.FA	IVW	3	0.65	0.48–0.88	5.57 × 10^–3^
UNC.FA	WM	3	0.63	0.42–0.94	2.42 × 10^–2^
UNC.FA	MR egger	3	4.93	0.004–5815.19	0.735

*MR, Mendelian randomization; IVW, inverse variance weighted; WM, white matter; FX, fornix (column and body of fornix); UNC, uncinate fasciculus; FA, fractional anisotropy.*

MR genetic instruments should only impact the outcome *via* exposure and not any other pathway (horizontal pleiotropy and heterogeneity). Therefore, we confirmed our results by applying sensitivity tests (Supplementary Tables 5, 6). The result showed little pleiotropy (FX.FA: *P* = 0.91 and UNC.FA: *P* = 0.67) and heterogeneity [FX.FA (IVW: *P* = 0.65; MR Egger: *P* = 0.76) and UNC.FA (IVW: *P* = 0.53; MR Egger: *P* = 0.70)] (Table 2). Further, MR-PRESSO demonstrated no significant horizontal pleiotropy of FX.FA (*P* = 0.762). The MR-PRESSO test could not be performed due to insufficient SNPs in UNC.FA. Additionally, the leave-one-out method confirmed the robustness of the causal association in FX.FA-Glaucoma and UNC.FA-Glaucoma pairs (Supplementary Figures 1A,B). Considering no heterogeneity or horizontal pleiotropy was present in the selected SNPs after sensitivity testing, the results of IVW were more reliable. Therefore, these results demonstrated that reducing fornix and uncinate fasciculus fractional anisotropy made a causal contribution to glaucoma.

**TABLE 2:** Sensitivity analysis between WM and glaucoma.

	Method	Q	*P*-value	Intercept	*P*-value
FX.FA	IVW	3.35	0.76	0.006	0.91
FX.FA	MR egger	3.33	0.65		
UNC.FA	IVW	0.71	0.70	−0.130	0.67
UNC.FA	MR egger	0.39	0.53		

*MR, Mendelian randomization; IVW, inverse variance weighted; WM, white matter; FX, fornix (column and body of fornix); UNC, uncinate fasciculus; FA, fractional anisotropy.*

### Causal Associations Between Region-of-Interest Volume and Glaucoma

**FIGURE 3:**
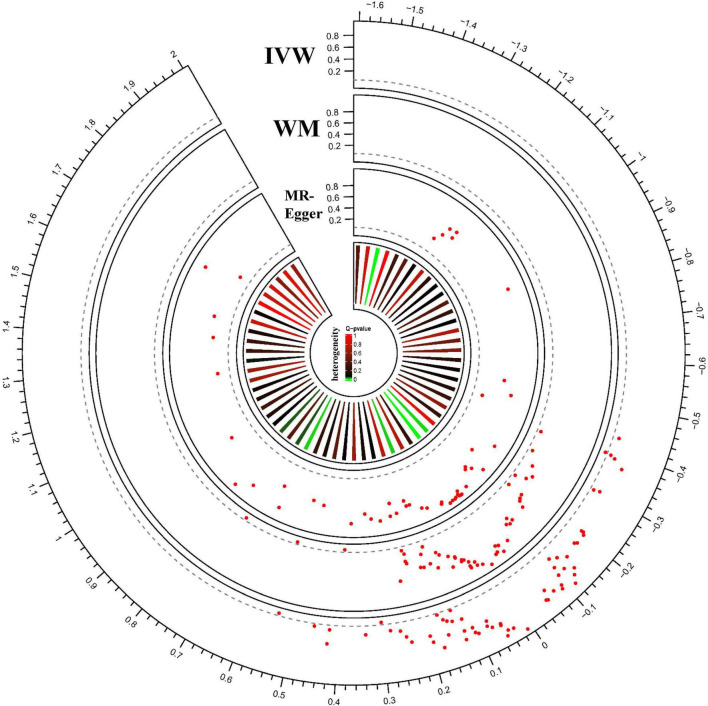
MR results for ROI-Glaucoma associations. Each radius represents one ROI measurement. From outer to inner layer: MR results analyzed with IVW; MR results analyzed with WM; MR results analyzed with MR-Egger; heterogeneity of each ROI measure. The red dot represents the ROI-Glaucoma relation. The X-axis shows the beta value, and Y-axis shows the *p*-value. The dotted gray line indicated the nominal p threshold (0.05).

By Mendelian randomization analysis, we found that the volumes of the right ventral diencephalon (right.Ventral.DC) and brain stem (Brain.stem) had a significant causal relationship with glaucoma (Table 3). The statistical results for the other regions are shown in Figure 3 and Supplementary Table 7. The results of IVW confirmed the volume of the right.ventral.DC (OR = 1.72, 95%CI = 1.17–2.52, *P* = 5.64 × 10^–3^) and Brain.stem (OR = 1.35, 95%CI = 1.08–1.69, *P* = 8.94 × 10^–3^) were positively associated with glaucoma risk. Meanwhile, weighted median (right.ventral.DC: *P* = 1.10 × 10^–3^; Brain.stem: *P* = 5.06 × 10^–3^) and MR-Egger (right.ventral.DC: *P* = 0.578; Brain.stem: *P* = 0.994). were used to verify the findings further.

**TABLE 3:** MR results between ROI and glaucoma.

ROI	Method	SNP	OR	95%CI	*P*
Right.ventral.DC	IVW	5	1.72	1.17–2.52	5.64 × 10^–3^
Right.ventral.DC	WM	5	2.07	1.34–3.21	1.10 × 10^–3^
Right.ventral.DC	MR egger	5	1.63	0.35–7.51	0.578
Brain.stem	IVW	13	1.35	1.08–1.69	8.94 × 10^–3^
Brain.stem	WM	13	1.48	1.13–1.95	5.06 × 10^–3^
Brain.stem	MR egger	13	1.00	0.31–3.20	0.994

*MR, Mendelian randomization; IVW, inverse variance weighted; WM, weighted median. Right.ventral.DC, right ventral diencephalon; brain.stem, Brain stem.*

We performed pleiotropic analysis (Supplementary Table 8) and heterogeneity tests (Supplementary Table 9). By calculating the MR-Egger intercept, no significant horizontal pleiotropy was observed in the right.ventral.DC (intercept = 0.003, *P* = 0.95) and Brain.stem (intercept = 0.018, *P* = 0.62) (Table 4). MR-PRESSO further validated the absence of significant horizontal pleiotropy (right.ventral.DC: *P* = 0.430; Brain.stem: *P* = 0.126). Besides, the results of heterogeneity tests showed that the right.ventral.DC (IVW: Q = 5.67, *P* = 0.22; MR-Egger: Q = 5.66, *P* = 0.13) and Brain.stem (IVW:Q = 17.92, *P* = 0.12); MR-Egger: Q = 17.51, *P* = 0.09) associated SNPs that were not significantly heterogeneous (Table 4). Meanwhile, the leave-one-out test confirmed the causal association in the right.ventral.DC-Glaucoma and Brain.stem-Glaucoma pairs (Supplementary Figures 1C,D). Therefore, the results of IVW were more reliable as no heterogeneity or horizontal pleiotropy was present in the selected SNPs after sensitivity testing. Thus the inferred causality between right.ventral.DC-glaucoma and Brain.stem-Glaucoma pairs were plausible.

**TABLE 4:** Sensitivity analysis between ROI and glaucoma.

	Method	Q	*P*	Intercept	*P*
Right.ventral.DC	IVW	5.67	0.22	0.003	0.95
Right.ventral.DC	MR egger	5.66	0.13		
Brain.stem	IVW	17.92	0.12	0.018	0.62
Brain.stem	MR egger	17.51	0.09		

*MR, Mendelian randomization; IVW, inverse variance weighted; Right.ventral.DC, right ventral diencephalon; Brain.stem, brain stem.*

## Discussion

To the best of our knowledge, this is the first MR analysis that leveraged population-scale human genetics to understand the causal relationship between neuroimaging polymorphisms and glaucoma in an unbiased manner. The imaging changes in the brain could not be directly associated as a cause of glaucoma in many current neuroimaging studies. However, our results show that the changes in the microstructures of the white matter and the volume of specific brain regions can affect the onset of glaucoma and reveal the existence of the brain-eye axis.

Large cohort studies have confirmed that patients with glaucoma have shown progressive vision loss even with a significant reduction in IOP (6). This finding indicates that IOP-independent mechanisms contribute to the etiopathogenesis of this disorder. Recently, there has been growing attention about the brain changes in glaucoma. In particular, numerous MRI studies performed on glaucoma patients have shown brain structural differences. Hernowo et al. (29) found decreased volume along the full length of the visual pathway for both gray and white matter in a follow-up study of glaucoma patients. Moreover, changes in the brain beyond the primary visual pathways, including the middle occipital gyrus, inferior temporal gyrus, the anterior thalamic radiation, corticospinal tract, superior temporal gyrus, and occipital lobe white matter, were also reported (30–32). However, the distinction between these brain changes as cause or consequence remains difficult as nearly all current literatures are cross-sectional with limited population and testing methods. Thus, understanding the brain-eye interactions across disease severity could provide valuable insights into the pathogenesis of glaucoma and lead to new treatment modalities.

MRI provides a non-invasive method to evaluate structural, metabolic, and functional changes in the brain. Diffusion-weighted imaging measures the 3-dimensional displacement of water molecules and provides important information about changes in the microstructures of the white matter (33). Many studies have used DTI to describe the white matter integrity in glaucoma. Hui et al. (34) reported decreased FA and increased MD at the optic nerve for the first time in a rat model of glaucoma. Decreased FA at the proximal portion of the optic nerve and chiasm were also detected in glaucoma patients (35, 36). Meanwhile, recent DTI studies demonstrated that glaucoma patients had a lower FA within the visual white matter pathway, negatively correlated with glaucoma severity (37). Additionally, the decreased FA in the inferior fronto-occipital fasciculus, the longitudinal and inferior frontal fasciculi, the putamen, the caudate nucleus, the anterior and posterior thalamic radiations, and the anterior and posterior limbs of the internal capsule were reported in glaucoma (38). In our results, decreased FX.FA and UNC.FA was a novel risk factor for glaucoma. Lower FA values indicate the presence of axonal disruption and reduced structural integrity. The fornix carries the axons projecting from the hippocampus, and fornix FA reduces in subjects with hippocampal atrophy, correlating with memory function and emotion regulation (39). Uncinate fascicles are the connecting fibers of the medial and lateral orbitofrontal gyrus, anterior cingulate gyrus, and anterior temporal lobe, including the amygdala and hippocampus. Lower FA in the uncinate fasciculus is involved in emotional regulation, including bipolar disorder and late-life depression (40, 41). Of note, glaucoma patients were associated with a reduction in cognition, memory, and psychological disorders such as depression and anxiety (12). Moreover, the hippocampal shrinks in glaucoma patients were associated with disease severity (42). Given this evidence, we speculate that the FA reduction in the fornix and uncinate fasciculus may precede glaucoma’s hippocampal atrophy and clinical symptoms.

Using a similar methodology, we detected the increased volumes of the right.ventral.DC and Brain. stem are both risk factors for glaucoma. Brain stem is involved in many of the basic functions of the encephalon, such as motor control and sensory analysis. Consistent with our results, Williams et al. (31). reported the volume of brain stem was larger in the early stage of glaucoma group and the volume decline later stage of the disease. The ventral diencephalon (VDC) is a group of structures including the hypothalamus, papillary body, subthalamic nucleus, substantia nigra, red nucleus, lateral geniculate nucleus (LGN), and medial geniculate nucleus (MGN) that cannot be normally distinguished by standard MRI images. Several primate studies have reported LGN input with additional modulatory input from various brain regions (including the striate cortex, the brain stem, and the thalamic reticular nucleus) (43). Moreover, recent observations with experimentally induced glaucoma have confirmed the loss of LGN neurons in the layers associated with the eye (44). Increased volume is likely a result of inflammatory processes and neuronal injuries, such as cellular swelling or microglial activation (31). Therefore, the injuries of right ventral diencephalon and stem brain may induce irreversible damage to the optic nerve in glaucoma.

Many research hypotheses that glaucoma originates in the brain but manifest as an eye disease. One of these studies indicated that glaucoma should be seen as part of a neurological disorder. They found that trans-lamina cribrosa pressure difference (TLCPD), calculated as the IOP minus the cerebrospinal fluid pressure, had a better association with glaucoma presence than IOP alone (45). Other studies have revealed that a higher proportion of AD patients had a probable diagnosis of glaucoma than controls, while the primary open-angle glaucoma (POAG) patients had a higher risk of developing AD (46, 47). RGCs are located in the retinal ganglion cell layer, and the axons of RGCs constitute the optic nerve and reach up to the LGN. Retrograde trans-synaptic degeneration is currently responsible for RGC loss in various neurodegeneration diseases, including Alzheimer’s (AD), and Parkinson’s (PD) disease (48). Meanwhile, various neuroimaging research converges evidence that glaucoma is associated with central nervous system changes and shares commonality with neurodegeneration diseases, including loss of RGCs and the deposition of abnormal proteins in specific anatomical areas like the hippocampus. The primary brain alterations could induce the RGCs death through retrograde trans-synaptic degeneration. Besides, recent studies also considered that glaucoma shares the common pathogenic mechanisms (oxidative stress, mitochondrial dysfunction, inflammation, and apoptosis) with neurodegeneration diseases (49). Our study conducted hypothesis-free, data-driven MR analyses and identified FA reduction in the fornix and uncinate fasciculus and increased volume in right.ventral.DC and brain stem are the primary causes of glaucoma. Therefore, combined with previous results, our data suggest that brain damage is an important factor in the onset of glaucoma. Future molecular and clinical research is required to improve our understanding of the underlying mechanisms. These potential pathogenic mechanisms linking brain alterations to retinal physiology will probably lead to early diagnosis of glaucoma with more appropriate and accurate approaches for its treatment.

Although more reliable conclusions can be obtained using large-scale GWAS studies, some limitations should be considered for the present study. First, data used in the current study were derived from the European population; therefore, the causal relationship between brain structural alteration and glaucoma in other populations such as Asia is unknown. Therefore, further research on different ethnicities should be conducted to explore the variation. Second, although we assessed the associations between brain alteration and glaucoma, it is expected that more MRI metrics of larger sample sizes can be used in future MR studies to find out the precise lesions in the brain of glaucoma patients. Third, the brain alteration in different types of glaucoma might be different, and further studies considering the glaucoma group are required. So forth, it was difficult to achieve a full non-overlap between exposure (DIT and ROI) and outcome (Glaucoma), even though we chose the data from publicly available summary data.

In summary, FA reduction in the fornix and uncinate fasciculus and increased volume of the right ventral diencephalon and brain stem might be the primary causes of glaucoma. Furthermore, our study illustrates that the fornix and uncinate fasciculus degenerations and injuries of the right ventral diencephalon and stem brain potentially increase the risk of glaucoma onset. These novel findings support the hypothesis that brain alteration can trigger glaucoma, and can be utilized for the advancement of diagnostics and therapies for glaucoma in the future.

## Data Availability Statement

The datasets presented in this study can be found in online repositories. The names of the repository/repositories and accession number(s) can be found in the article/Supplementary Material.

## Ethics Statement

Ethical review and approval was not required for the study on human participants in accordance with the local legislation and institutional requirements. Written informed consent for participation was not required for this study in accordance with the national legislation and the institutional requirements.

## Author Contributions

JZ: conceptualization and writing—review and editing. PW: methodology. KL: investigation and writing—original draft. BC: resources. RY and YC: supervision. All authors contributed to the article and approved the submitted version.

## Conflict of Interest

The authors declare that the research was conducted in the absence of any commercial or financial relationships that could be construed as a potential conflict of interest.

## Publisher’s Note

All claims expressed in this article are solely those of the authors and do not necessarily represent those of their affiliated organizations, or those of the publisher, the editors and the reviewers. Any product that may be evaluated in this article, or claim that may be made by its manufacturer, is not guaranteed or endorsed by the publisher.

## Footnotes

1
http://stnava.github.io/ANTs/

## Supplementary Material

Supplementary Figure 1 Leave-one-out permutation analysis for the fornix fractional anisotropy **(A)**, uncinate fasciculus fractional anisotropy **(B)**, and the volume of right ventral diencephalon **(C)** and brain stem **(D)**.
