# Supplementary figures and images for: Implicating Causal Brain Magnetic Resonance Imaging in Glaucoma Using Mendelian Randomization
Source: Front Med (Lausanne). 2022 Jul 1;9:956339. doi: 10.3389/fmed.2022.956339 (PMC9283577; doi:10.3389/fmed.2022.956339)

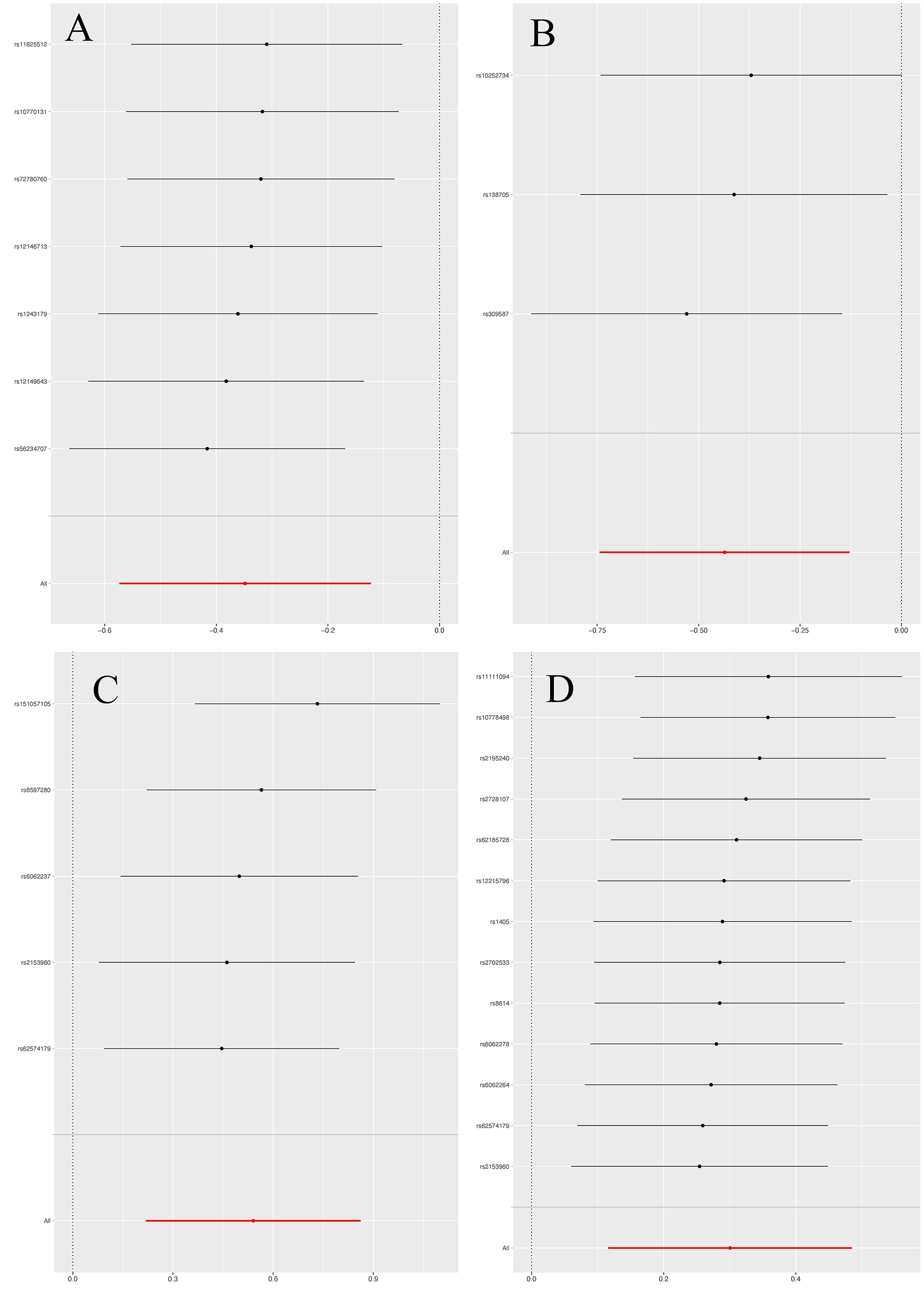

Supplement: Supplementary Figure 1 — Leave-one-out permutation analysis for the fornix fractional anisotropy (A), uncinate fasciculus fractional anisotropy (B), and the volume of right ventral diencephalon (C) and brain stem (D). [file Image_1.tif]
